# Cost-effectiveness of alternate strategies for childhood immunization against meningococcal disease with monovalent and quadrivalent conjugate vaccines in Canada

**DOI:** 10.1371/journal.pone.0175721

**Published:** 2017-05-04

**Authors:** Thomas E. Delea, Derek Weycker, Mark Atwood, Dion Neame, Fabián P. Alvarez, Evelyn Forget, Joanne M. Langley, Ayman Chit

**Affiliations:** 1 Policy Analysis Inc. (PAI), Brookline, MA, United States of America; 2 Sanofi Pasteur, Toronto, ON, Canada; 3 Sanofi Pasteur, Lyon, France; 4 Department of Community Health Sciences, Faculty of Medicine, University of Manitoba, Winnipeg, MB, Canada; 5 Canadian Center for Vaccinology and the Departments of Pediatrics and Community Health and Epidemiology, Dalhousie University, Halifax, NS, Canada; 6 Sanofi Pasteur, Swiftwater, PA, United States of America; 7 Lesli Dan Faculty of Pharmacy, University of Toronto, Toronto, ON, Canada; University of Cambridge, UNITED KINGDOM

## Abstract

**Background:**

Public health programs to prevent invasive meningococcal disease (IMD) with monovalent serogroup C meningococcal conjugate vaccine (MCV-C) and quadrivalent meningococcal conjugate vaccines (MCV-4) in infancy and adolescence vary across Canadian provinces. This study evaluated the cost-effectiveness of various vaccination strategies against IMD using current and anticipated future pricing and recent epidemiology.

**Methods:**

A cohort model was developed to estimate the clinical burden and costs (CAN$2014) of IMD in the Canadian population over a 100-year time horizon for three strategies: (1) MCV-C in infants and adolescents (MCV-C/C); (2) MCV-C in infants and MCV-4 in adolescents (MCV-C/4); and (3) MCV-4 in infants (2 doses) and adolescents (MCV-4/4). The source for IMD incidence was Canadian surveillance data. The effectiveness of MCV-C was based on published literature. The effectiveness of MCV-4 against all vaccination regimens was assumed to be the same as for MCV-C regimens against serogroup C. Herd effects were estimated by calibration to estimates reported in prior analyses. Costs were from published sources. Vaccines prices were projected to decline over time reflecting historical procurement trends.

**Results:**

Over the modeling horizon there are a projected 11,438 IMD cases and 1,195 IMD deaths with MCV-C/C; expected total costs are $597.5 million. MCV-C/4 is projected to reduce cases of IMD by 1,826 (16%) and IMD deaths by 161 (13%). Vaccination costs are increased by $32 million but direct and indirect IMD costs are projected to be reduced by $46 million. MCV-C/4 is therefore dominant vs. MCV-C/C in the base case. Cost-effectiveness of MCV-4/4 was $111,286 per QALY gained versus MCV-C/4 (2575/206 IMD cases/deaths prevented; incremental costs $68 million).

**Conclusions:**

If historical trends in Canadian vaccines prices continue, use of MCV-4 instead of MCV-C in adolescents may be cost-effective. From an economic perspective, switching to MCV-4 as the adolescent booster should be considered.

## Introduction

Invasive meningococcal disease (IMD) is a life-threatening illness caused by the bacterium *Neisseria meningitidis*, and occurs when bacteria spread to the meninges (the membranes enveloping the central nervous system), the bloodstream, or both. Case fatality rates are about 10% even with early acute care, and survivors may suffer severe and permanent disabilities [1].

As in most developed countries, IMD is uncommon in Canada. Between 2001 and 2005, almost all Canadian provinces and territories except Nunavut introduced monovalent conjugate meningococcal vaccines against serogroup C (MCV-C) into the routine immunization schedule. The use of these vaccines has helped control illness due to that serogroup [2]. Serogroup B accounted for 55% of all cases during the 2002–2011 period, with serogroups C (19%), Y (17%), and W-135 (5%) occurring at lower frequencies[3]. The annual incidence of IMD in all Canadians was 0.6 per 100,000 during 2005–2010, and was highest among children aged <5 years (7.0 and 1.8 per 100,000 children aged <1 and 1–4 years, respectively) and adolescents (1.2 per 100,000 children aged 15–19 years) [4].

In Canada, three MCV-C and three quadrivalent meningococcal conjugate vaccines against serogroups A, C, Y, and W-135 (MCV-4) are available [5]. A vaccine for meningococcal B disease also has been approved. The Canadian National Advisory Committee on Immunization (NACI) recommends that healthy children be immunized with MCV-C routinely at 12 months of age and a routine adolescent booster dose at around 12 years of age with either MCV-C or MCV-4, depending on local epidemiology and other programmatic considerations [6]. All provinces and territories offer infant MCV-C programs and a booster dose in adolescence. Seven of the authorities use MCV-C in their public programs for adolescents and the remaining six use MCV-4 [4]. No jurisdiction has incorporated vaccination against meningococcal B disease in its routine programs.

De Wals and colleagues evaluated the cost-effectiveness of adolescent vaccination against IMD with MCV-C or MCV-4 in Canada in 2007 [7]. Results of that study suggested that adolescent vaccination with MCV-C may be cost-saving and that adolescent vaccination with MCV-4 may be cost-effective compared with adolescent vaccination with MCV-C. However, the vaccine prices that were used in this study are now a decade old, and costs of vaccines against meningococcal disease in Canada have declined over time, likely as a consequence of the tender process by which vaccines are purchased by the provinces. Further, guidelines for the economic evaluation of health technologies suggest that it may be appropriate to account for anticipated future changes in drug prices in these evaluations [8]. This study was undertaken to determine if there is an incremental value of MCV-4 vs MCV-C by estimating the cost-effectiveness of MCV-4 vs MCV-C for the infant and/or adolescent dose in Canada, using recent epidemiologic and vaccine pricing data, and incorporating projections of future vaccine prices based on observed historical trends.

## Materials and methods

### Model description

A multi-cohort Markov model was developed in Microsoft Excel® to estimate the expected clinical and economic impact of vaccination against IMD in a hypothetical prevalent cohort of 33.3 million Canadian population and subsequent birth cohorts of 385 thousand newborns each year over a 100 year time horizon. Vaccine strategies considered in this analysis included (1) MCV-C vaccination in infants (12 months) and adolescents (13 years) (MCV-C/C); (2) MCV-C vaccination in infants and MCV-4 vaccination in adolescents (MCV-C/4); and (3) MCV-4 vaccination in infants and adolescents (MCV-4/4). In Canada, three MCV-4 vaccines are available (Menactra [Sanofi Pasteur Ltd.], Menveo [Novartis Vaccines and Diagnostics Inc.], Nimenrix [GlaxoSmithKline]), although only two (Menactra and Menveo) are being used in public health programs at the time of this analysis (Nimenrix did not win any market share on the Canadian tender despite it being available). This analysis uses effectiveness information for Menactra, under the assumption it would be similar for Menveo.

The population is characterized based on age (in one-month increments for persons aged <2 years, and one-year increments thereafter) [9]. In each cycle, the model projects the number of IMD cases by serogroup based on population size, age- and serogroup-specific disease rates, vaccine coverage, and vaccine effectiveness (including direct and indirect or “herd” effects). For persons developing IMD, the model projects the clinical consequences of IMD in terms of deaths and long-term sequelae [7, 10, 11]. All persons are assigned age-specific utility values. Those developing sequelae are assumed to have lower health-state utilities than those who do not.

The reduction in risk of infection in vaccinated persons (i.e., direct effects) is assumed to depend on the vaccine received (MCV-C or MCV-4), age at immunization, and time since immunization. ISPOR-SMDM Modeling Good Research Practices recommend the use of dynamic transmission models to estimate indirect effects where vaccination may effect transmission of the disease[12]. In the model, indirect effects for vaccinated and unvaccinated persons are estimated using an adaptation of a mathematical approximation of dynamic transmission modeling for use in cohort models [13, 14]. With this approach, the infection rate in each year of the projection for all age-groups (*i* = 1 to *N*) is multiplied by a scaling factor (*s*) that is calculated as follows:

$$s=\sum_{i=1}^{N}(1-P_{i}e_{i})\;w_{i}$$

Where *P*_*i*_ is the percent of persons in each age group who are vaccinated, *e*_*i*_ is the effectiveness of the vaccine in each group (reflecting the direct effects of vaccination and vaccine coverage), and *w*_*i*_ is a weight reflecting the percent of the entire cohort within each age group. The scalar for the herd effect was calibrated so that indirect effects in the model would approximately match those reported in the aforementioned economic evaluation of MCV-4 vaccination in Quebec by De Wals [7]. In this study, De Wals reported the numbers of cases with no vaccination, as well as the reduction in cases due to direct and indirect effects associated with MCV-C/C and MCV-C/4 vaccination. De Wals did not report results by age, so calibration was conducted for the overall population. We estimated the scalar for the herd effect by setting all model inputs to match those employed by De Wals, then solving for the value of the parameter that yielded an estimate of the number of residual cases with MCV-C/4 vaccination that matched that reported by De Wals (0.90 per million). This calibration model yielded a projection of the percentage reduction in incidence due to direct and herd effects for MCV-C/4 vs. MCV-C/C that is somewhat greater than that reported by De Wals (58% vs. 50%). However, the percent of the total reduction in incidence due to herd effects was less with our model than reported by De Wals (71% vs. 85%).

For each vaccine strategy, the model was used to calculate the number of IMD clinical cases, case fatalities, non-fatal cases developing sequelae, life years (LYs) and quality-adjusted life years (QALYs) lost due to IMD, as well as the expected costs of vaccination (vaccine, administration, adverse events), public health response, and the total direct and indirect costs of treatment of IMD. Future costs and health benefits were discounted at 5% annually [8]. A societal perspective was employed.

### Model estimation

Model parameters were estimated from a variety of sources and are described below and summarized in Table 1.

**Table 1:** Key model parameter values.

Parameter	Age (Years)					Distribution	Sources
	<1	1	2–4	5–17	≥18		
No. of Persons	384,282	384,282	1,152,847	5,060,115	28,169,866	N/A	Statistics Canada
IMD case rates by serogroup, per 100K							
C	1.306	0.56	0.143	0.13	0.065	Beta	IMPACT 2011
Y	1.903	0.815	0.208	0.19	0.094	Beta	IMPACT 2011
W135	0.718	0.308	0.079	0.072	0.036	Beta	IMPACT 2011
IMD case-fatality rates by serogroup, per 100							
C	6.3	6.3	6.3	6.3	15.4	Beta	IMPACT 2011
Y	5.5	5.5	5.5	5.5	11.4	Beta	IMPACT 2011
W135	4	4	4	4	14.7	Beta	IMPACT 2011
Sequelae rates, per 100*	13.2	13.2	13.2	13.2	20	Uniform	Stovall 2002, Erickson 2001
Vaccine coverage, %	—	91	—	84	—	N/A	Public Health Ontario 2013
Vaccine effectiveness, 1 yr. post-receipt, %							
MCV-C	—	91.7	91.7	93.0**	93	Uniform	De Wals (2011), Cohn (2013)
MCV-4	—	91.7	91.7	93.0**	93	Uniform	
Vaccine waning, annual, %							
MCV-C	—	7.93	7.93	13.66	13.66	Uniform	De Wals (2011), Cohn (2013)
MCV-4	—	7.93	7.93	13.66	13.66	Uniform	
Utilities							
Age-specific values	0.98	0.98	0.98	0.98–0.96	0.96–0.59	Uniform	Caro (2007)
Disutility from sequelae	0.28	0.28	0.28	0.28	0.27	Uniform	De Wals (2007)
Direct medical and indirect costs, CAN$							
Vaccine							
Price per dose, initial***							
MCV-4	—	31.42	31.42	31.42	31.42	N/A	PHAC / Assumption
MCV-C	—	13.12	13.12	13.12	13.12	N/A	PHAC / Assumption
Administration, per dose	—	5.07	5.07	5.07	11.5	N/A	De Wals (2007)
Adverse events, per dose	—	0.07	0.07	0.07	0.07	N/A	De Wals (2007)
Direct Medical							
IMD, per case							
Treatment	14,144	14,144	14,144	14,144	14,144	Uniform	De Wals (2007)
Public health response	4,250	4,250	4,250	4,250	4,250	Uniform	Ortega-Sanchez (2008)
Sequelae, annual, per case	19,124	19,124	19,124	19,124	4,085	Uniform	De Wals (2007)
Indirect							
Short-term indirect costs, per case	3,047	3,047	3,047	3,047	3,047	Uniform	De Wals (2007)
Workforce participation, %	0	0	0	13.2	66	N/A	Statistics Canada
Average wage, annual	0	0	0	3,494	46,171	N/A	Statistics Canada
Productivity loss from sequelae, %	46.8	46.8	46.8	46.8	68.4	Uniform	De Wals (2007)

N/A: not applicable; IMD: invasive meningococcal disease; MCV-C: meningococcal conjugate vaccine—serogroup C; MCV-4: meningococcal conjugate vaccine—quadrivalent (serogroups A, C, Y, W135)

*Sequelae include skin scarring, single/multiple amputation, hearing loss, neurologic disability; rate per 100 IMD survivors

**Higher effectiveness assumed for adolescents

***The price of MCV-4 assumed to remain constant at $31.42 through 2016 and decline at 15.6% annually thereafter. The price of MCV-C assumed to remain constant at $13.12 through 2015 and decline at 10.0% annually thereafter.

All costs and benefits discounted at 5% annually

#### IMD rates

Age- and serogroup-specific rates of IMD for unvaccinated persons, assuming no herd effects from MCV-4 vaccination, were estimated by combining data on the number of IMD cases in Canada from the National Enhanced IMD Surveillance System [15], with unpublished data on the distributions of IMD cases by age and serogroup from Canada’s Immunization Monitoring Program ACTive (IMPACT) Network, and age-specific population estimates [9]. Because the focus of the present study is on the incremental impact of using MCV-4 in place of MCV-C, data on the number of cases were limited to the 3-year period prior to widespread use of MCV-4 (i.e., 2007–2009). From point estimates derived using these sources, linear interpolation and extrapolation was used to project values for all ages in monthly increments until the age of 2 years, and annually thereafter. Cases with an unknown serogroup were allocated proportionally across those with known serogroups. Estimated IMD rates were adjusted upward by 10% to account for potential under-reporting of IMD cases [16], but no upward adjustment was made for potential under-diagnosis of IMD as was done in the cost-effectiveness analysis by De Wals and colleagues [7]. Disease due to serogroup A was not included as no cases were reported during the surveillance period. Further details on these calculations may be found in the online supplement.

#### IMD case-fatality and sequelae rates

Age- and serogroup-specific IMD case-fatality rates were based on unpublished data from the IMPACT Network. The rate of sequelae among children surviving the initial event (13%) was based on the incidence of ≥1 long-term sequelae—including skin scarring, amputation, hearing loss, neurologic disability—among 136 children in Arkansas aged <21 years who developed IMD and survived the initial acute phase [17]. For adults, the rate of sequelae (20%) was based on the incidence of permanent physical sequelae—including amputation, hearing loss, and skin scarring—in a study of complications of IMD in college students in Allegheny County, Pennsylvania, from 1990 to 1999 [18]. These rates are consistent with those used in the cost-effectiveness analysis by De Wals and colleagues [7], as well in analyses by Caro and colleagues [19].

#### Vaccine effectiveness

MCV-C effectiveness in the first year following immunization for infants (91.7%) was based on data for children aged 1 year during the 2 years post-vaccination following an outbreak of serogroup C meningococcal disease and the subsequent implementation of a routine immunization program in Quebec, Canada [20]. Because this study did not report effectiveness for adolescents (only for children ≥2 years of age at vaccination), effectiveness for adolescents (93%) was based on the assumed effectiveness used in a cost-effectiveness analysis (CEA) reported by the Advisory Committee on Immunization Practices (ACIP) of the U.S. Centers for Disease Control and Prevention (CDC) in their recommendations for the prevention and control of meningococcal disease [21]. Because data on effectiveness of MCV-4 in Canada were unavailable at the time this analysis was conducted, effectiveness of MCV-4 against all serogroups was assumed to be the same as for MCV-C, consistent with the approach used by De Wals and colleagues [7].

**Fig 1:**
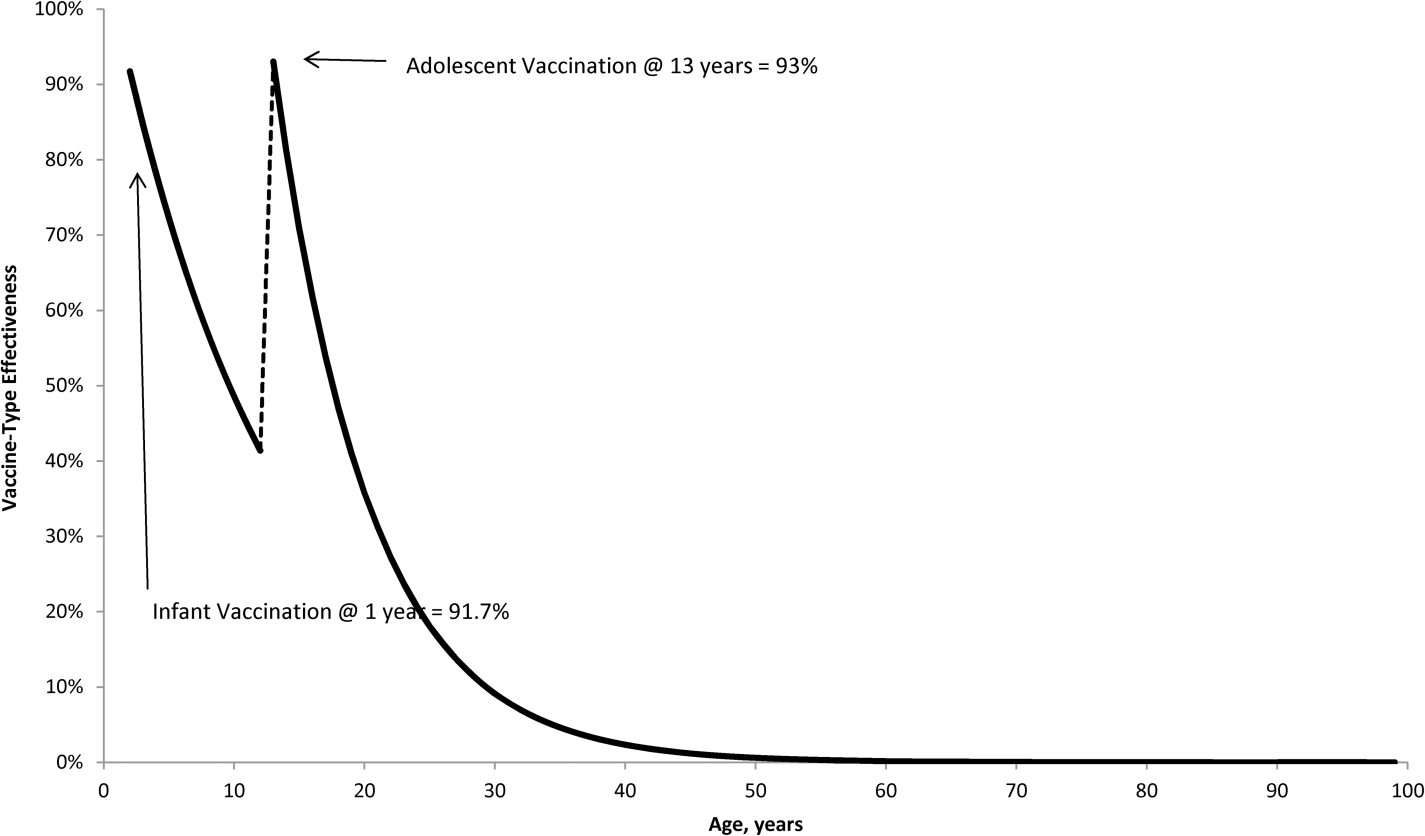
Vaccine effectiveness by age.

Vaccine effectiveness was assumed to wane over time (Fig 1). For infants, waning was estimated to be 7.93% annually by fitting an exponential distribution to the point estimates for children aged 1 year (66.7% [-38 to 92]) and children aged ≥2 years (87.8% [67.3 to 95.4]), respectively, during the period ≥2 years from vaccination in the Quebec study [20]. Waning for adolescents was estimated to be 13.66% annually by fitting an exponential curve to immunogenicity point estimates ≥2 years post vaccination reported by the ACIP [21]. Coverage with MCV-C or MCV-4 was assumed to be 91% among infants (age = 12 months) and 84% among adolescents (age = 13 years) [22].

#### Vaccine costs

**Fig 2:**
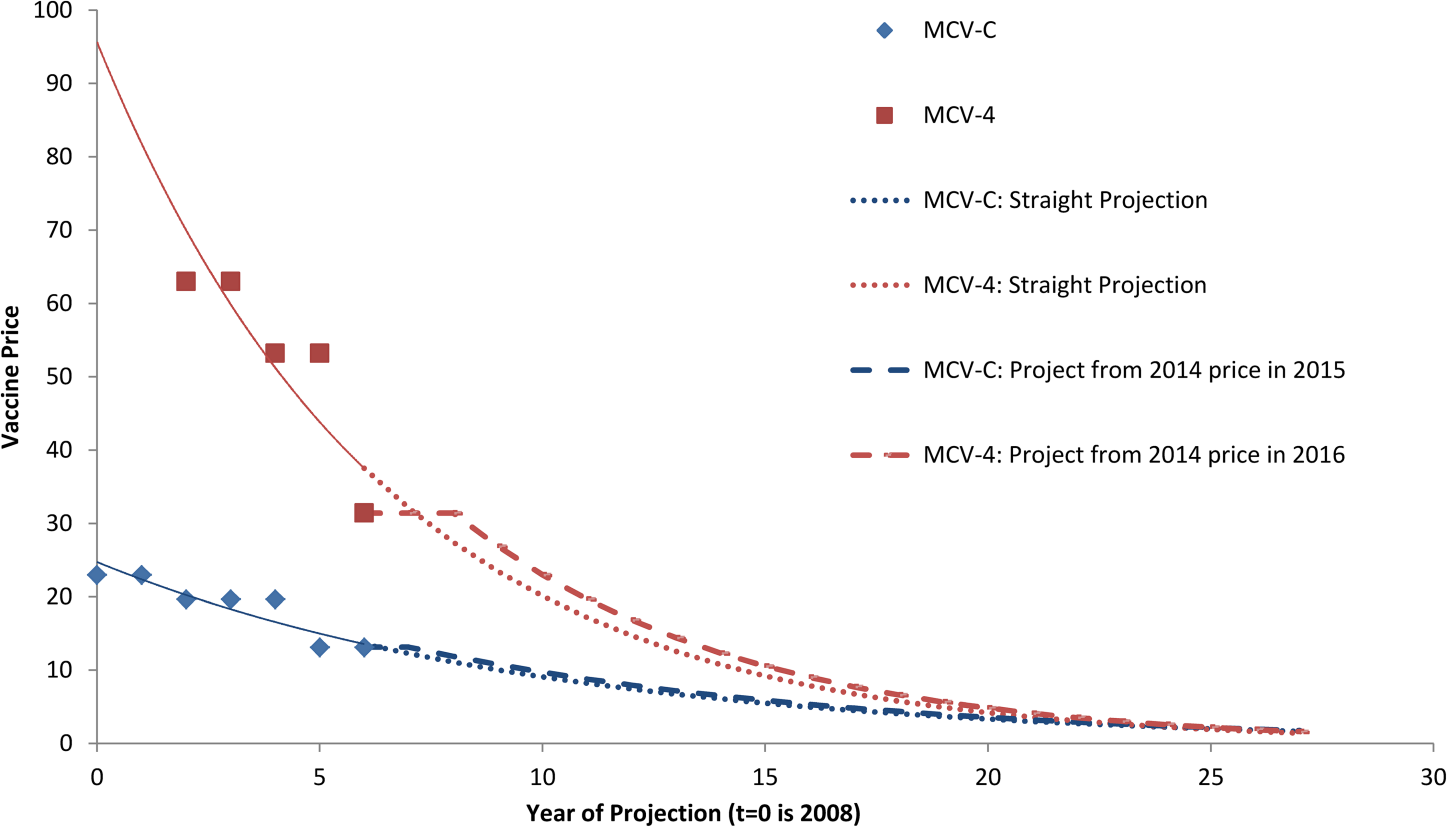
Historical and projected vaccine price.

In Canada, the public (federal/provincial/territorial) bulk procurement program purchases vaccines through a national tender that is renewed every 3 years. The costs of vaccines to the program have declined over time (Unpublished data. Public Health Agency of Canada) (Fig 2). To reflect the likely continuation of this trend during the projection period, vaccine prices in each year of the projection were estimated by fitting exponential curves to historical data on vaccine prices in past tender allocations. Because tenders typically last 2–3 years, the price of MCV-4 was assumed to remain constant at its current price ($31.42) through 2016 and decline (based on the exponential projection) at 15.6% annually thereafter. The price of MCV-C was assumed to remain constant at its current price ($13.12) through 2015 and decline (based on the exponential projection) at 10.0% annually thereafter. The price of MCV-4 was assumed to not fall below that of MCV-C. Costs of vaccine administration and vaccine-related adverse events were based on data from De Wals *et al*. [7]. For costing purposes, it was assumed that two doses of MCV-4 would be required to confer protection of infants, consistent with dosing recommendations for Menactra and Menveo in unvaccinated children 9–23 and 7–23 months of age, respectively [23, 24].

#### Direct and indirect costs of IMD

The direct medical costs of treating IMD and the sequelae of IMD were based on data from De Wals *et al*. [7]. The cost of public health response was based on a US cost-effectiveness analysis of meningococcal vaccination [10]. Costs in US dollars were converted to Canadian dollars based on health-related purchasing power parity [25]. Indirect costs—including those for morbidity-related work loss from IMD and sequelae of IMD, and mortality-related work loss from premature death—were estimated using a human-capital approach based on Canadian labor force survey estimates and census data and estimates of productivity loss from published literature [7]. All costs were adjusted to 2014 Canadian dollars [26].

#### Utility values

QALYs were calculated by combining estimates of life expectancy and the incidence of IMD sequelae with utility values. Utility values (QALY weights) are cardinal values anchored on 1.0 for perfect health and 0.0 for death that represent individuals’ preferences for different health outcomes. Age-specific utility values were from a prior study of the cost-effectiveness of IMD vaccination in the United States [19]. Decrements in utility values associated with IMD sequelae were based on EuroQol (EQ-5D) utility index values from the aforementioned study of complications of IMD in college students in Allegheny County, Pennsylvania [18].

### Analyses

#### Base-case analyses

For each comparison (MCV-C/4 vs. MCV-C/C, MCV-4/4 vs. MCV-C/C, and MCV-4/4 vs. MCV-C/4), the differences in clinical and economic outcomes were calculated using base-case estimates of model inputs. Cost-effectiveness for each comparison was calculated in terms of cost per LY gained and cost per QALY gained. Strategies with lower costs and greater effectiveness were considered “dominant”. When assessing whether a vaccination strategy was cost-effective, threshold values of $56,000 and $168,000 per QALY gained were used, which correspond to threshold values for “highly cost-effective” (<gross domestic product [GPD] per capita) and cost effective (<3 times GDP per capita), respectively, in the World Health Organization CHOosing Interventions that are Cost-Effective (WHO-CHOICE) guidelines [27, 28].

#### Sensitivity analyses

One-way deterministic sensitivity analyses were conducted to assess the robustness of findings with respect to clinically and economically reasonable changes in key parameter estimates. Probabilistic sensitivity analyses accounting for uncertainty surrounding key model parameters (1,000 replications) were used to generate credible intervals (95%) for measures of interest as well as scatter plots for incremental costs and QALYs and acceptability curves.

## Results

### Base-case analyses

Assuming MCV-C vaccination in infants and adolescents, the annual incidence of IMD in Canada is projected to be approximately 0.39 per 100,000 persons annually throughout the projection period, reflecting no change in the current vaccination program. With MCV-C vaccination in infants and MCV-4 vaccination in adolescents, incidence is projected to decline asymptotically from 0.39 per 100,000 in year 1 to approximately 0.25 per 100,000 in year 30 (after which it remains relatively constant). With MCV-4 vaccination in both infants and adolescents, incidence declines asymptotically to approximately 0.16 per 100,000 in year 50. The projected gradual decline in incidence with the MCV-4 vaccination programs reflects both the increasing proportion of persons in the population who are vaccinated as well as the increasing effects of herd immunity.

Over the 100 year projection period, assuming MCV-C vaccination in infants and adolescents, there would be a total of 11,438 IMD cases, 1,715 cases of IMD sequelae, and 1,195 IMD deaths in Canada. LYs and QALYs lost due to IMD would be 36,612 and 30,122, respectively (discounted: 4,238 and 3,633, respectively) (Table 2). Total national expected costs (discounted) would be $597.5 million, including $182 million in vaccination costs, $81 million in direct medical costs, and $335 million in indirect costs.

**Table 2:** Expected lifetime clinical and economic outcomes under alternative strategies for vaccination against meningococcal disease in Canada.

	MCV-C/C	MCV-C/4	MCV-4/4
Clinical outcomes (mean, 95% CI)			
No. of IMD cases	11,438 (2,030–41,092)	9,612 (1,778–34,061)	7,037 (999–27,653)
No. of IMD sequelae cases	1,715 (244–6,757)	1,448 (207–5,934)	1,086 (120–4,485)
No. of IMD deaths	1,195 (95–5,374)	1,034 (82–4,620)	828 (50–3,921)
Life-years Lost (vs. No IMD)			
Not discounted	36,612 (3,899–141,891)	31,531 (3,453–122,542)	24,545 (2,024–101,965)
Discounted	4,238 (377–17,927)	3,806 (347–16,148)	3,171 (213–14,021)
QALYs lost (vs. No IMD)			
Not discounted	30,122 (3,555–113,511)	25,831 (3,079–96,592)	19,803 (1,819–79,774)
Discounted	3,633 (365–14,462)	3,253 (335–13,023)	2,677 (208–11,287)
Costs, discounted, CAN$ millions (mean, 95% CI)			
Direct medical			
IMD	36.2 (6.0–131.3)	32.0 (5.6–109.6)	25.2 (3.4–97.2)
Public health response	10.9 (1.9–38.6)	9.7 (1.7–32.7)	7.6 (1.0–27.5)
Sequelae	33.5 (5.9–125.5)	30.0 (5.3–110.8)	23.6 (3.4–92.7)
Total direct medical costs	80.7 (14.4–290.9)	71.7 (13.3–248.3)	56.3 (8.3–213.5)
Vaccination			
Vaccine	63.76 (63.76–63.76)	95.40 (95.40–95.40)	198.47 (198.47–198.47)
Administration	117.25 (117.25–117.25)	117.25 (117.25–117.25)	154.23 (154.23–154.23)
Adverse events	0.96 (0.96–0.96)	0.96 (0.96–0.96)	0.96 (0.96–0.96)
Total vaccination costs	181.97 (181.97–181.97)	213.62 (213.62–213.62)	353.66 (353.66–353.66)
Indirect	334.9 (39.6–1,275.8)	298.3 (35.8–1,117.9)	241.6 (24.3–971.0)
Total			
Direct medical + vaccination	262.7 (196.4–472.9)	285.3 (226.9–461.9)	410.0 (362.0–567.1)
Direct medical + indirect	415.6 (59.1–1,549.3)	369.9 (54.5–1,375.8)	298.0 (33.7–1,188.6)
Direct medical + vaccination + indirect	597.5 (241.1–1,731.3)	583.6 (268.2–1,589.4)	651.6 (387.3–1,542.3)

MCV-C: meningococcal conjugate vaccine—serogroup C; MCV-4: meningococcal conjugate vaccine—quadrivalent (serogroups A, C, Y, W135); IMD: invasive meningococcal disease; LY: life-years; QALY: quality-adjusted life-years; 95%CI: 95% credible interval (based on 2.5%tile and 97.5% of second order simulations)

Adolescent vaccination with MCV-4 in place of MCV-C is projected to reduce the number of IMD cases by 1,826 (16%), cases of IMD sequelae by 267 (16%), and IMD deaths by 161 (13%). LYs and QALYs lost due to IMD would be reduced by 5,081 (14%) and 4,291 (14%), respectively (432 and 380 discounted, respectively). Total costs are projected to be reduced by $14 million (2%), reflecting increased vaccination costs of $32 million, which are offset by savings of $9 million in direct medical costs and savings of $37 million in indirect costs.

Compared with infant and adolescent vaccination with MCV-C, infant and adolescent vaccination with MCV-4 is projected to reduce the number of IMD cases by 4,401 (46%), cases of IMD sequelae by 629 (43%), and IMD deaths by 367 (35%). LYs and QALYs lost due to IMD would be reduced by 12,067 (38%) and 10,319 (40%), respectively (1,067 and 956 discounted, respectively). Total costs are projected to *increase* by $54 million (9%), reflecting increased vaccination costs of $172 million, which are only partly offset by savings of $24 million in direct medical costs and savings of $93 million in indirect costs.

Compared with infant vaccination with MCV-C and adolescent vaccination with MCV-4, MCV-4 for both infants and adolescents is projected to reduce the number of IMD cases by 2,575 (37%), cases of IMD sequelae by 362 (33%), and IMD deaths by 206 (25%). LYs and QALYs lost due to IMD would be reduced by 6,986 (28%) and 6,028 (30%), respectively (635 and 576 discounted, respectively). Total costs are projected to increase by $68 million (10%), reflecting increased vaccination costs of $140 million, which are only partly offset by savings of $15 million in direct medical costs and savings of $57 million in indirect costs.

Because MCV-C/4 is projected to have lower costs and yield more QALYs than MCV-C/C, MCV-C/4 is considered to be “dominant” versus MCV-C/C in the base case (Table 3). Because MCV-C/4 dominates MCV-C/C, it is appropriate to compare MCV-4/4 vs MCV-C/4. The cost-effectiveness of MCV-4/4 versus MCV-C/4 is projected to be $111,286 per QALY gained. Based on WHO-CHOICE thresholds for cost-effectiveness [27], MCV-4/4 would be a cost-effective use of healthcare resources since this amount falls within the range of 1–3 times the Canadian GDP per capita [28].

**Table 3:** Cost-effectiveness of alternative strategies for vaccination against meningococcal disease in Canada.

Measure	MCV-C/4 vs. MCV-C/C	MCV-4/4 vs. MCV-C/C	MCV- 4/4 vs. MCV-C/4
Incremental costs (95% CI), CAN$ millions	-14.0	54.1	68.1
	(-200.3–28.9)	(-272.5–154.7)	(-84.1–127.6)
Incremental LYs (95% CI)	431	1,067	636
	(18–2,180)	(101–4,458)	(74–2,399)
Incremental QALYs (95% CI)	380	955	575
	(17–1,926)	(101–3,807)	(73–2,035)
Incremental cost per LY gained (95% CI), CAN$	Dominant	41,401	100,170
	(Dominant—106,568)	(20,338–251,917)	(57,563–450,627)
Incremental cost per QALY gained (95% CI), CAN$	Dominant	46,534	111,286
	(Dominant—129,502)	(25,075–305,155)	(70,566–543,647)

LYs: Life years; CI: Confidence interval; QALYs: Quality-adjusted life years. Dominant: Strategy is less costly and more effective than comparator.

MCV-C: meningococcal conjugate vaccine—serogroup C; MCV-4: meningococcal conjugate vaccine—quadrivalent (serogroups A, C, Y, W135); LY: life-years; QALY: quality-adjusted life years; ICER: incremental cost-effectiveness ratio; 95%CI: 95% credible interval (based on 2.5%tile and 97.5% of second order simulations)

### Sensitivity analyses

Estimates of the cost-effectiveness of MCV-C/4 were found to be relatively insensitive to changes in parameter values and assumptions, remaining dominant compared to MCV-C/C in all scenarios except when: (1) The IMD case rate was reduced by 50% (cost per QALY gained = $30,439); (2) the lower bound of the 95% confidence interval for vaccine effectiveness was used (cost per QALY gained = $54,592); (3) it was assumed there were no indirect effects with vaccination (cost per QALY gained = $137,303); and (4) prices were assumed to remain at current levels (cost per QALY gained = $181,404) (Table 4). Cost-effectiveness of MCV-4/4 vs MCV-C/4 was sensitive to changes in IMD incidence and case-fatality rates, vaccine effectiveness, the assumption of herd effects, the assumption of declining prices, the discount rate, and the model time frame. MCV-C/4 remained dominant compared with MCV-C/C even under the assumption that there would be no herd effects for five years after the change in the vaccination program. MCV-C/4 also remained dominant compared with MCV-C/C under the assumption that (1) vaccine prices would follow a step-function corresponding to 3 year tenders, (2) the average annual rate of decline in prices would be the same for MCV-C/4 as for MCV-C/C, and (3) prices would never fall below $4 per vaccine (the price that Sanofi currently charges UNICEF for MCV-4 and below which industrial capacity to manufacturer the vaccines might not be sustainable).

**Table 4:** Sensitivity analyses on cost-effectiveness (cost per QALY) of alternative strategies for vaccination against meningococcal disease in Canada.

	MCV-C/4 vs MCV-C/C			MCV-4/4					
				MCV-C/C			MCV-C/4		
	ΔCosts (CAN$ Millions)	ΔQALYs	ICER (CAN$)	ΔCosts (CAN$ Millions)	ΔQALYs	ICER (CAN$)	ΔCosts (CAN$ Millions)	ΔQALYs	ICER (CAN$)
Basecase	-18.6	427	Dominant	47.6	1,022	46,534	66.2	595	111,286
IMD case rate									
Lower by 50%	6.5	214	30,439	109.6	511	214,526	103.1	297	346,704
Higher by 50%	-43.8	641	Dominant	-14.5	1,533	Dominant	29.3	892	32,814
IMD case-fatality rate									
Lower by 40%	-6.3	262	Dominant	78.2	628	124,566	84.4	365	231,043
Higher by 40%	-31.0	592	Dominant	16.9	1,416	11,953	48.0	824	58,187
IMD sequelae rate									
Lower by 10%	-17.5	426	Dominant	50.2	1,019	49,320	67.8	593	114,332
Higher by 10%	-19.7	428	Dominant	44.9	1,025	43,767	64.6	597	108,260
Vaccine coverage									
Lower by 10% (81%/74%)	-16.8	377	Dominant	40.7	913	44,541	57.5	536	107,220
Higher by 10% (100%/94%)	-20.2	476	Dominant	54.1	1,123	48,174	74.3	647	114,744
Vaccine effectiveness in first year									
Lower bound of 95% CI (60.1%/39.0%)	10.0	183	54,592	95.8	616	155,527	85.8	433	198,237
Upper bound of 95% CI (98.3%/99.0%)	-21.7	454	Dominant	40.3	1,083	37,220	62.0	630	98,553
Waning of vaccine effectiveness									
Lower by 10% (7.1%/12.3%)	-21.9	456	Dominant	43.3	1,062	40,781	65.2	605	107,743
Higher by 10% (8.7%/15.0%)	-15.8	402	Dominant	51.3	988	51,914	67.1	586	114,546
Herd effects with MCV-4*									
Delayed by 5 years	-9.5	354	Dominant	64.7	882	73,302	74.2	528	140,368
None	15.8	115	137,303	108.8	443	245,879	93.0	327	284,173
Vaccine prices									
Stepped/equal rate of decline, $4 minimum	-10.1	427	Dominant	82.0	1,022	80,283	92.1	595	154,857
Remain at current levels	77.5	427	181,404	403.3	1,022	394,627	325.8	595	547,726
Utilities									
Lower by 25%	-18.6	324	Dominant	47.6	775	61,384	66.2	451	146,767
Higher by 25%	-18.6	485	Dominant	47.6	1,147	41,476	66.2	662	100,023
Medical Care Costs									
Lower by 25%	-17.4	427	Dominant	50.6	1,022	49,490	68.0	595	114,341
Higher by 25%	-19.8	427	Dominant	44.5	1,022	43,578	64.4	595	108,232
Two infant doses of MCV-C	-18.6	427	Dominant	-22.6	1,022	Dominant	-4.0	595	Dominant
Discount rates									
0%	-240.7	4,787	Dominant	-288.2	10,935	Dominant	-47.6	6,148	Dominant
3%	-49.8	946	Dominant	4.0	2,216	1,817	53.8	1,269	42,396
Modeling horizon									
20 years	-26.8	361	Dominant	46.1	785	58,698	72.8	424	171,886
40 years	-22.0	407	Dominant	47.3	937	50,538	69.3	530	130,723
60 years	-17.7	417	Dominant	52.0	989	52,569	69.7	572	121,905
80 years	-16.8	427	Dominant	52.1	1,022	50,995	68.9	595	115,851

ΔCost: Incremental costs; ΔQALY: Incremental quality-adjusted life-years; ICER: Incremental cost per QALY gained. Dominant: Strategy is less costly and more effective than comparator. CI: Confidence interval; MCV-C: meningococcal conjugate vaccine—serogroup C; MCV-4: meningococcal conjugate vaccine—quadrivalent (serogroups A, C, Y, W135); IMD: invasive meningococcal disease

*Serogroups A, Y, W135

To further assess the sensitivity of model results to projections of the price of MCV-4, a threshold analysis was conducted to identify the price of MCV-4 at which the each of the vaccine strategies would be cost-effective given the threshold ICERs of $56,000 and $168,000 per QALY gained, respectively (1 and 3 times GDP per capita, respectively). In this analysis, the prices of both vaccines were held constant over time at their initial values. Assuming an ICER threshold of $56,000 per QALY gained, MCV-C/4 is projected to be cost-effective compared with MCV-C/C if the price of MCV-4 is less than or equal to $23.75. With an ICER threshold of $168,000 per QALY gained, MCV-C/4 is projected to be cost-effective compared with MCV-C/C if the price of MCV-4 is less than or equal to $30.60. These prices represent premiums of $10.63 and $17.48, respectively, over the estimated current price of MCV-C ($13.12). For the comparison of MCV-4/4 versus MCVC/C, the prices at which the former is cost-effective relative to the latter given ICER thresholds of $56,000 and $168,000 per QALY gained are $15.38 and $20.68, respectively (premiums of $2.26 and $7.56, respectively). The prices of MCV-4 at which MCV-4/4 is cost-effective compared with MCV-C/4 at these thresholds are $11.37 and $15.94, respectively. Thus, with a threshold ICER of $56,000 per QALY gained, the price of MCV-4 would need to be less than that of MCV-C for MCV-4/4 to be cost-effective compared with MCV-C/4.

In probabilistic sensitivity analyses, MCV-C/4 was more costly and more effective than MCV-C/C in 64% of the simulations and more effective and less costly (i.e., dominant) in 36% of the simulations. MCV-C/4 was cost-effective versus MCV-C/C in 47% of simulations at a threshold value of $56,000 per QALY gained (1 x GDP per capita) and in 65% of simulations at a threshold value of $168,000 per QALY gained (3 x GDP per capita). Compared with MCV-C/4, MCV-4/4 was more costly and more effective in 90% of simulations; it was more effective and less costly (i.e., dominant) in 10% of simulations. The probability that MCV-4/4 is cost-effective versus MCV-C/4 was 22% at a threshold value of $56,000 per QALY gained and 45% at a threshold value of $168,000 per QALY gained. Scatter plots and acceptability curves for all comparisons based on probabilistic sensitivity analyses are included in the Online Supplement.

## Discussion

Results of these analyses suggest that using MCV-4 in lieu of MCV-C for the adolescent booster in Canada, following universal meningococcal C vaccination in infancy, may be cost effective. Although replacing infant MCV-C vaccination with infant MCV-4 vaccination was cost-effective in base-case analyses, in probabilistic sensitivity analyses the probability that such a strategy would be cost effective was less than 50%.

In the only comparison that is the same in both our study and the aforementioned analysis by De Wals and colleagues (MCV-C/4 vs. MCV-C/C), estimated cost-effectiveness was substantially lower in our study (dominant) versus theirs ($113,206 per QALY gained) [7]. While there are a number of reasons for this difference, the most significant one is different vaccine prices used in the two analyses. Other sources of the difference are the modeling approach employed (prevalent plus successive birth cohorts in this study vs. prevalent cohort in De Wals), rates of incidence of IMD (lower in this study), and case-fatality rates (lower in this study).

Two important features of our analysis should be noted. First, we assumed in base-case analyses that the price of MCV-4 would approach that of MCV-C over time, based on exponential projections fit to historical data on from prices from prior tenders. While we believe this assumption to be reasonable, and consistent with recommendations for the conduct of economics evaluations in Canada and elsewhere, results are less favorable for MCV-4 vaccination if vaccine prices are assumed to remain at their current levels throughout the projection period.

Second, we estimated the indirect effects of MCV-4 vaccination on transmission using a modification of an approach first set forth by Bauch and colleagues [13, 14]. With this method, the benefits of herd effects are assumed to be the same across age groups. Nasopharyngeal carriage of *N*. *meningitidis* is highest in adolescents and young adults, who serve as reservoirs for transmission of the disease [29]. Accordingly, we may have overestimated the herd effects associated with infant MCV-4 vaccination. It also should be noted that although this general approach for approximating dynamic transmission models using a cohort model was developed for the evaluation of vaccination of influenza [13, 14], it should be generalizable to vaccination of other diseases.

Limitations of our study should be noted. First, because data on the epidemiology of IMD were not available for each province/territory separately, we used estimates of IMD rates and associated case-fatality rates based on data from all of Canada. To the extent that there are systematic differences across provinces/territories in disease epidemiology—as well as other parameters such as vaccine prices and medical costs—caution should be used in generalizing from the results of this study to a particular province/territory.

Second, initial effectiveness of infant vaccination against IMD was based on a single epidemiologic study of the vaccination experience in Quebec, Canada over a seven-year period of time [20]. This epidemiologic study employed the screening method to estimate vaccine effectiveness, which is less reliable than the case-control method. However, point estimates from this study are largely consistent with those from the various studies of the vaccination experience in the UK and Spain [30–32].

Third, because the Quebec study did not report effectiveness for adolescents, the initial effectiveness of adolescent vaccination (93%) was based on the estimate used by the ACIP of the U.S. CDC in their CEA of adolescent vaccination [21]. This estimate is conservative relative to the reported effectiveness of vaccination of children ≥2 years of age reported in the aforementioned Quebec study (97%) [20].

Fourth, absent comparable data on the effectiveness of MCV-4, it was assumed that effectiveness of this vaccine against all serogroups would be the same as that for MCV-C against serogroup C. However, data from randomized controlled trials suggest that immunogenicity of MCV-4 is non-inferior to MCV-C, and prior cost effectiveness evaluations have assumed similar effectiveness for MCV-4 and MCV-C[7, 33]. Further, results of sensitivity analyses suggest that MCV-C/4 may be cost-effective (cost per QALY gained less than one times GDP per capita) even under conservative assumptions regarding vaccine effectiveness. Data from a recent published data from Ontario reported that there was a significant decrease in the incidence of serogroup Y cases in the three years following the introduction of adolescent MCV-4 vaccination (19.8% reduction per year) [34]. It is difficult to draw firm conclusions from these data, however, as only three years of data following the initiation of the MCV4 program were available.

In England and Wales, the incidence of IMD has been declining for more than a decade, but meningococcal group W (MenW) cases have been increasing since 2009 due to rapid endemic expansion of a single clone belonging to cc11 that is associated with severe disease with unusual clinical presentations [35]. The analyses reported herein may be conservative as they did not consider the potential effects of future changes in underlying IMD incidence rates that might be associated with the potential transmission of such MenW clones to the Canadian setting.

Estimates of the costs of IMD were based on the best available published estimates, which may not be reflective of current treatment and costs. Future research may benefit from primary cost studies to document the current costs of IMD in Canada and elsewhere.

Projections of vaccines prices were based on a limited set of data and are therefore associated with substantial uncertainty. Given the small numbers of observations, we believe the assumption of a constant rate of decline in prices is not unreasonable and that fitting a more complicated price function with a variable rate of decline would be infeasible. While it is true that there are tender periods, and that in reality the curve will be a "step function", the precise dates of the future tenders are not known, and the use of a smooth function doesn’t materially impact the results. Also, while it is true that the price of MCV-4 is lower than MCV-3 by 20 years, the absolute difference beyond that point is not material (effectively representing an assumption of equal long-term prices).Last, in assessing cost-effectiveness, we use a threshold range from $56,000 to $168,000 per QALY gained, which corresponds to values for “highly cost-effective” and “cost-effective” in WHO-CHOICE guidelines (based on GDP per capita) [27, 28]. There are no recent published threshold values for evaluation of vaccines in Canada, and the applicability of the WHO thresholds, in particular the higher value of $168,000 per QALY gained, in Canada is debatable. Rocchi and colleagues reviewed the drug reimbursement recommendations of the advisory board of the Common Drug Review from September 2003 to March 2007, and reported that medications with a positive recommendation had cost-effectiveness ratios as high as $80,000 per QALY gained [36]. However, these recommendations were made a decade or more ago, and also take into consideration the quality of the clinical evidence, as well as the cost-effectiveness ratios, and therefore do not provide an unambiguous representation of the threshold. Using a threshold value of $100,000 per QALY gained, the estimated probability that MCV-C/4 would be cost-effective compared with MCV-C/C would be only 56%, and the base case estimate of the ICER for MCV-4/4 vs. MCV-C/4 would no longer be below the threshold.

In summary, results of this study suggest that if historical trends in vaccine prices continue, the use of MCV-4 rather than MCV-C in adolescents may be cost effective. Canadian provinces without MCV-4 programs should consider adoption of programs that include MCV-4 vaccination for adolescents.

## Supporting information

S1 Table Derivation of IMD incidence rates by serogroup. (DOCX)

S2 Table Derivation of IMD incidence rates by age and serogroup. (DOCX)

S1 Fig Scatter plot of difference in expected costs versus difference in expected quality-adjusted life-years for alternative strategies for vaccination against meningococcal disease in Canada. (TIFF)

S2 Fig Acceptability curves for cost-effectiveness (cost per QALY) for alternative strategies for vaccination against meningococcal disease in Canada. (TIF)
