# Supplementary material for: Cost-effectiveness of alternate strategies for childhood immunization against meningococcal disease with monovalent and quadrivalent conjugate vaccines in Canada
Source: PLoS One. 2017 May 4;12(5):e0175721. doi: 10.1371/journal.pone.0175721 (PMC5417484; doi:10.1371/journal.pone.0175721)
Supplement: S1 Table — (DOCX) [file pone.0175721.s001.docx]

| **S1 Table. Derivation of IMD incidence rates by serogroup** |  |  |  |  |  |
| --- | --- | --- | --- | --- | --- |
| **Estimate** | **IMD Cases, by Year** | | | | **Source** |
|  | **2007** | **2008** | **2009** | **Average** |  |
| Number of IMD cases in Canada | 229 | 195 | 209 | 211 | Public Health Agency of Canada, IMD (2013) |
| Number of IMD cases from IMPACT, by serogroup |  |  |  |  | IMPACT Final Report (12/31/2011) |
| A | 0 | 0 | 0 | 0.0 |  |
| B | 68 | 46 | 47 | 53.7 |  |
| C | 18 | 15 | 6 | 13.0 |  |
| Y | 19 | 19 | 16 | 18.0 |  |
| W135 | 9 | 7 | 5 | 7.0 |  |
| Unknown | 5 | 1 | 4 | 3.3 |  |
| Total | 119 | 88 | 78 | 95 |  |
| Distribution of IMD casesfrom IMPACT, by serogroup (C, Y and W135 only)* |  |  |  |  | Calculated |
| C | 59.6% | 52.9% | 63.5% | 58.5% |  |
| Y | 15.8% | 17.2% | 8.1% | 14.2% |  |
| W135 | 16.7% | 21.8% | 21.6% | 19.6% |  |
| Estimated number of IMD cases in Canada, by serotype |  |  |  |  | Calculated |
| C | 136.6 | 103.1 | 132.7 | 123.5 |  |
| Y | 36.2 | 33.6 | 16.9 | 29.9 |  |
| W135 | 38.2 | 42.6 | 45.2 | 41.4 |  |
| *Assuming "unknown" serotype distributed proportionally among known serotypes | | | |  |  |
