# Supplementary material for: Cost-effectiveness of alternate strategies for childhood immunization against meningococcal disease with monovalent and quadrivalent conjugate vaccines in Canada
Source: PLoS One. 2017 May 4;12(5):e0175721. doi: 10.1371/journal.pone.0175721 (PMC5417484; doi:10.1371/journal.pone.0175721)
Supplement: S2 Table — (DOCX) [file pone.0175721.s002.docx]

| **S2 Table. Derivation of IMD incidence rates by age and serogroup** | | | | | | | | |  |  |  |  |  |  |  |  |  |  |  |  |
| --- | --- | --- | --- | --- | --- | --- | --- | --- | --- | --- | --- | --- | --- | --- | --- | --- | --- | --- | --- | --- |
|  | **IMD Cases, by Year^1^** | | | | |  | **IMD Cases, by Serogroup (C, Y, and W135)^2^** | | |  | **Canadian Population^3^** | |  | **IMD Rates (per 100K), by Serogroup (C, Y, and W135)^2^** | | |  | **IMD Rates (per 100K), by Serogroup (C, Y, and W135), Adjusted for 10% Underreporting^2^** | | |
| **Age** | **2007** | **2008** | **2009** | **Average^1^** | |  |  |  |  |  |  |  |  |  |  |  |  |  |  |  |
|  |  |  |  | **No.** | **%** |  | **C** | **Y** | **W135** |  | **Thousands** | **%** |  | **C** | **Y** | **W135** |  | **C** | **Y** | **W135** |
| <9 mos. | 9 | 11 | 15 | 11.7 | 12.2% |  | 15.12 | 3.66 | 5.07 |  | 269.47 | 0.81% |  | 5.6101 | 1.3590 | 1.8816 |  | 1.4512 | 2.1141 | 0.7983 |
| 9-11 mos. | 3 | 3 | 1 | 2.3 | 2.4% |  | 3.02 | 0.73 | 1.01 |  | 89.82 | 0.30% |  | 3.3662 | 0.8154 | 1.1290 |  | 0.8707 | 1.2684 | 0.4790 |
| 12-23 mos. | 7 | 4 | 7 | 6.0 | 6.3% |  | 7.77 | 1.88 | 2.61 |  | 359.3 | 1.10% |  | 2.1638 | 0.5242 | 0.7258 |  | 0.5598 | 0.8154 | 0.3079 |
| 2-10 yrs. | 17 | 12 | 13 | 14.0 | 14.7% |  | 18.14 | 4.39 | 6.08 |  | 3,279.32 | 9.90% |  | 0.5532 | 0.1340 | 0.1855 |  | 0.1431 | 0.2085 | 0.0787 |
| 11-14 yrs. | 4 | 7 | 2 | 4.3 | 4.5% |  | 5.62 | 1.36 | 1.88 |  | 1,625.65 | 4.90% |  | 0.3454 | 0.0837 | 0.1158 |  | 0.0894 | 0.1302 | 0.0491 |
| 15-18 yrs. | 18 | 3 | 4 | 8.3 | 8.7% |  | 10.80 | 2.62 | 3.62 |  | 1,797.29 | 5.40% |  | 0.6008 | 0.1455 | 0.2015 |  | 0.1554 | 0.2264 | 0.0855 |
| >=19 yrs. | 61 | 48 | 37 | 48.7 | 51.0% |  | 63.06 | 15.28 | 21.15 |  | 25,833.23 | 77.70% |  | 0.2441 | 0.0591 | 0.0819 |  | 0.0631 | 0.0920 | 0.0347 |
|  |  |  |  |  |  |  |  |  |  |  |  |  |  |  |  |  |  |  |  |  |
| Total | 119 | 88 | 79 | 95.3 | 100.0% |  | 123.53 | 29.92 | 41.43 |  | 33,254.09 | 100.00% |  | 0.3715 | 0.0900 | 0.1246 |  | 0.0961 | 0.1400 | 0.0529 |
| ^1^ IMPACT Final Report (12/31/2011) | | | |  |  |  |  |  |  |  |  |  |  |  |  |  |  |  |  |  |
| ^2^ Calculated | |  |  |  |  |  |  |  |  |  |  |  |  |  |  |  |  |  |  |  |
| ^3^ Statistic Canada (2012) | | |  |  |  |  |  |  |  |  |  |  |  |  |  |  |  |  |  |  |
